# Supplementary material for: Probenecid Inhibits Respiratory Syncytial Virus (RSV) Replication
Source: Viruses. 2022 Apr 27;14(5):912. doi: 10.3390/v14050912 (PMC9147281; doi:10.3390/v14050912)
Supplement: Supplementary file 1 [file viruses-14-00912-s001.zip › viruses-1682509-supplementary.pdf]

viruses

MDPI

Supplementary Materials

Probenecid Inhibits Respiratory Syncytial Virus (RSV) Replication

Jackelyn Murray [1], Harrison C. Bergeron [1], Les P. Jones [1], Zachary Beau Reener[1], David E. Martin [2], Fred D. Sancilio [3] and Ralph A. Tripp [1,2,*]

Table S1. OAT3 Expression.

HEp-2 Cells	Probenecid Treated	DMSO only
	Raw Ct Value	Raw Ct Value
ACTB	16.3 (95% CI 14.8-17.8)	15.3 (95% CI 14.4-16.2)
OAT3	No Ct	32.1 (95% CI 31.2-33)
BALB/c Mice	Probenecid Treated (200 mg/kg)	
	Fold change reduction	
OAT3	15.6 (95% CI 3.8-27.4)	
Paired Ct Values	Probenecid Treated	PBS
	Raw Ct Value	Raw Ct Value
ACTB	11.94	11.10
OAT3	33.40	31.26
ACTB	11.66	11.65
OAT3	33.65	30.38
ACTB	11.91	11.70
OAT3	36.06	30.72
ACTB	14.5	13
OAT3	37	31

HEp-2 cells were treated with IC_{90} concentration of probenecid [7.2uM] or mock treated (DMSO only) for 24h. OAT3 transcripts were determined as described in Methods. Since probenecid treatment resulted in undetectable levels of OAT3 transcripts, fold-change was not performed. Lung RNA was extracted at day 2 pi in mice treated with 200 mg/kg probenecid or PBS (i.e., 24h post treatment). OAT3 gene expression in 200 mg/kg probenecid treated mice was normalized to a housekeeping gene, ACTB (the gene for beta (β)-actin) which is an endogenous control and compared to PBS treated mice. The data is represented as the reciprocal of $2^{\Delta\Delta Ct}$ (fold-change decrease). Mean paired Ct values are included for ACTB and OAT3. Data represent the mean Ct values or fold-change of three individual experiments and 95% confidence intervals (CI).

Table S2. BAL cells.

A. BAL cells/mL.

Sex	Group	BAL cells/mL + (SD)					
		Day 3		Day 5		Day 7	
Males	Prophylactic 2mg/kg	1.25E+05	*3.23E+04*	1.19E+05	*3.06E+04*	1.31E+04	*3.75E+03*
	Prophylactic 200mg/kg	2.44E+05	*9.91E+04*	2.94E+05	*5.00E+04*	1.31E+04	*5.15E+03*
	Treatment 2mg/kg	1.94E+05	*8.91E+04*	1.20E+05	*5.18E+04*	1.75E+04	*9.84E+03*
	Treatment 200mg/kg	2.65E+05	*8.48E+04*	1.45E+05	*5.18E+04*	1.94E+04	*8.51E+03*
	PBS	2.44E+05	*2.39E+04*	1.00E+05	*3.06E+04*	1.95E+04	*7.98E+03*
Females	Prophylactic 2mg/kg	2.69E+05	*1.32E+05*	2.56E+05	*5.76E+04*	7.50E+04	*1.22E+05*
	Prophylactic 200mg/kg	3.08E+05	*1.26E+05*	2.80E+05	*6.75E+04*	1.25E+05	*7.71E+04*
	Treatment 2mg/kg	3.60E+05	*1.40E+05*	2.70E+05	*6.94E+04*	1.35E+05	*8.91E+04*
	Treatment 200mg/kg	3.13E+05	*1.39E+05*	1.40E+05	*1.16E+05*	9.17E+04	*3.79E+04*
	PBS	2.35E+05	*1.01E+05*	1.70E+05	*1.02E+05*	2.50E+05	*1.05E+05*

B. BAL cells types.

**Sex**	**DPI**	**Cell Type**	**Total BAL cells (*SD*)**									
			Prophylactic 2mg/kg		Prophylactic 200mg/kg		Treatment 2mg/kg		Treatment 200mg/kg		PBS	
**Males**	**3**	Macrophages	3.06E+04	*7.12E+03*	7.05E+04	*5.65E+04*	3.79E+04	*1.67E+04*	4.22E+04	*1.45E+04*	4.87E+04	*5.36E+03*
		Lymphocytes	5.67E+02	*4.93E+02*	1.38E+03	*1.04E+03*	7.88E+02	*5.17E+02*	9.63E+02	*4.85E+02*	4.67E+02	*4.51E+02*
		Neutrophils	4.00E+02	*1.00E+02*	1.08E+03	*1.37E+03*	0.00E+00	*0.00E+00*	6.25E+02	*2.63E+02*	1.83E+02	*3.18E+02*
		Eosinophils	1.33E+02	*2.31E+02*	8.00E+01	*1.79E+02*	1.38E+02	*1.89E+02*	0.00E+00	*0.00E+00*	6.50E+02	*2.18E+02*
	5	Macrophages	2.15E+04	*1.16E+04*	5.66E+04	*3.39E+04*	2.66E+04	*1.30E+04*	2.92E+04	*1.16E+04*	1.92E+04	*6.21E+03*
		Lymphocytes	3.90E+02	*3.75E+02*	1.25E+03	*7.58E+02*	3.63E+02	*1.25E+02*	2.38E+02	*2.10E+02*	4.60E+02	*1.95E+02*
		Neutrophils	0.00E+00	*0.00E+00*	4.63E+02	*8.28E+02*	4.25E+02	*2.50E+02*	4.63E+02	*3.13E+02*	2.10E+02	*1.52E+02*
		Eosinophils	8.00E+01	*1.00E+02*	4.38E+02	*5.06E+02*	1.13E+02	*1.65E+02*	1.50E+02	*1.60E+02*	1.00E+02	*9.35E+01*
	7	Macrophages	1.66E+04	*1.13E+04*	1.27E+04	*5.10E+03*	1.66E+04	*1.08E+04*	1.75E+04	*7.41E+03*	1.86E+04	*7.92E+03*
		Lymphocytes	6.75E+02	*5.44E+02*	2.06E+02	*1.18E+02*	4.50E+02	*4.22E+02*	1.45E+03	*1.83E+03*	6.30E+02	*3.93E+02*

		Neutrophils	3.00E+01	*7.50E+01*	4.38E+01	*8.75E+01*	8.13E+01	*1.63E+02*	6.88E+01	*1.38E+02*	2.00E+01	*4.47E+01*
		Eosinophils	0.00E+00	*0.00E+00*	0.00E+00	*0.00E+00*	0.00E+00	*0.00E+00*	0.00E+00	*0.00E+00*	0.00E+00	*0.00E+00*
Females	3	Macrophages	6.89E+04	*4.04E+04*	7.71E+04	*2.67E+04*	4.40E+04	*2.24E+04*	5.71E+04	*3.56E+04*	5.11E+04	*2.66E+04*
		Lymphocytes	2.38E+03	*1.77E+03*	2.10E+03	*1.27E+03*	4.88E+02	*5.39E+02*	1.20E+03	*7.55E+02*	1.35E+03	*1.03E+03*
		Neutrophils	1.30E+02	*0.00E+00*	3.00E+02	*4.24E+02*	4.88E+02	*8.78E+02*	1.13E+03	*1.30E+03*	6.00E+02	*8.22E+02*
		Eosinophils	5.40E+02	*5.34E+02*	5.00E+02	*7.07E+02*	0.00E+00	*0.00E+00*	6.00E+02	*3.77E+02*	0.00E+00	*0.00E+00*
	5	Macrophages	5.22E+04	*2.11E+04*	5.49E+04	*3.23E+04*	3.45E+04	*2.11E+04*	3.05E+04	*8.33E+03*	5.18E+04	*1.96E+04*
		Lymphocytes	1.45E+03	*7.68E+02*	8.50E+02	*3.66E+02*	1.20E+03	*1.04E+03*	4.63E+02	*3.04E+02*	1.80E+03	*1.07E+03*
		Neutrophils	0.00E+00	*0.00E+00*	9.00E+01	*2.01E+02*	3.75E+02	*6.24E+02*	7.50E+01	*1.50E+02*	2.80E+02	*3.90E+02*
		Eosinophils	3.30E+02	*4.50E+02*	1.70E+02	*3.80E+02*	1.63E+02	*3.25E+02*	2.50E+02	*3.79E+02*	1.60E+02	*3.58E+02*
	7	Macrophages	2.64E+04	*1.38E+04*	2.42E+04	*2.44E+04*	5.37E+04	*1.99E+04*	1.24E+04	*1.03E+04*	1.36E+04	*4.05E+03*

Lymphocytes	4.00E+02	*3.11E+02*	2.60E+02	*2.88E+02*	6.88E+02	*3.42E+02*	1.50E+02	*1.73E+02*	2.50E+02	*3.24E+02*
Neutrophils	1.00E+02	*2.50E+02*	3.70E+02	*5.81E+02*	1.38E+02	*2.75E+02*	0.00E+00	*0.00E+00*	1.30E+02	*1.30E+02*
Eosinophils	1.20E+02	*2.50E+02*	2.10E+02	*2.79E+02*	4.75E+02	*5.62E+02*	0.00E+00	*0.00E+00*	3.00E+01	*6.71E+01*

BAL cells were collected from male and female BALB/c mice (n>3/group) at days 3, 5, or 7 post-infection. (A) Cells were enumerated by hemocytometer and Trypan blue and presented as BAL cells/mL (+SD; italics). (B) A portion of BAL cells were cytospun onto glass slides, fixed with 4% paraformaldehyde, and stained with hematoxylin and eosin. At least 100 cells per slide were phenotyped based on morphology, and values represent total number of each cell type in the BAL (+SD, in italics). There were no significant differences between treatment groups compared to PBS control as determined by two-way ANOVA.
